# Development of ECG Monitoring System and Implantable Device with Wireless Charging

**DOI:** 10.3390/mi10010038

**Published:** 2019-01-08

**Authors:** Jae-Ho Lee, Dong-Wook Seo

**Affiliations:** 1 Electronic and Telecommunications Research Institute, Daegu 42994, Korea; jhlee1229@etri.re.kr; 2 Department of Radio Communication Engineering, Korea Maritime and Ocean University, Busan 49112, Korea

**Keywords:** electrocardiogram (ECG), implantable device, MedRadio, wireless charging, wireless power transfer

## Abstract

We developed an implantable electrocardiogram (ECG) monitoring system and demonstrated its performance through an in vivo test. In the system, the implantable device senses not only the ECG signal of the animal but also the voltage level of the secondary cell and temperature inside the implantable device, and users can check the transmitted information through a PC program or a mobile application. The adoption of wireless charging technology eliminates the use of a lead wire and repetitive surgery to replace the implantable device. The proposed wireless charging technology demonstrated experimentally a wireless power transfer efficiency of approximately 30%. To minimize the size of the implantable device, the antenna and coil were integrated into a size of 34 mm × 14 mm. Communication between the implantable device and the basestation can reach up to 2.4 m when the implantable device is inserted into a porcine skin sample.

## 1. Introduction

As the fourth industrial revolution has accelerated, Internet of Things (IoT) technologies have spread deeply into human lives. In the early stage, IoT technologies were mainly applied to home appliances as additional convenience features. Recently, the application of IoT technology has extended beyond the simple control of objects to the sensing of human bio-signals.

As bio-signals are generally very weak, biosensors should have contact with the human body. Electrocardiogram (ECG) sensors used to monitor arrhythmia have been developed primarily as patch types [1,2]. Recently, many of the emerging sensors such as electric, optic, and chemical sensors have been introduced to sense various physiological signals on the epidemies [3]. However, existing patch-type ECG sensors should be attached to the human body, causing serious inconvenience to people in need of service. For this reason, many biosensors have evolved into implant or remote sensing devices. Remote sensing devices have been mainly developed using radar technologies, which sense changes in periodic motions such as heart rate and respiratory function [4,5]. On the other hand, the implantable devices directly sense the bio-signals inside the human body and transfer the sensed signals to a device outside the body. The implantable devices are mainly powered by the primary cells. However, the primary cells have a limited life, and thus additional surgery is required to replace dead cells on a regular basis. To relieve these problems, wireless power transfer (WPT) technology has been applied to devices that are implanted into shallow organs through the skin [6,7,8,9,10,11,12]. The functions of the implantable devices can be largely classified into three categories: Monitoring, treatment, and auxiliary. First, monitoring devices sense and observe the patient’s vital signals [5,6]; a typical example of such a device is the loop recorder which records heartbeats. Second, examples of therapeutic implantable devices include pacemakers and dorsal stimulators [7,8,9]. These devices directly drive an electrical stimulation to organs such as the heart or nerve. Finally, auxiliary implantable devices include cochlear implants and retinal implants. They change external sounds or lights to electrical signals and deliver the changed signals to the auditory or optic nerves [11,12]. Therapeutic or auxiliary implantable devices are limited to people suffering from special diseases or handicaps, whereas monitoring implantable devices can be applied to an ordinary person. In particular, implant monitoring systems can be effective for people living in areas where immediate medical services are not available.

Most of the state-of-art implantable devices work only when powered wirelessly from out-body [13]. Previous ECG implantable devices also work in a similar way [5,6]. For small animals, there is no problem even if the implantable device operates only during wireless power transmission. However, for patients with a chronic disease, inadequate mobility, or arrhythmia, it is necessary to continuously monitor vital signs 24/7. In this case, it is virtually impossible to continuously supply power from the outside. To solve this problem, in this paper, we adopt WPT technology to wirelessly charge the secondary cell in an implantable device. This solution can continue to operate the implantable device until the secondary cell is discharged. Additionally, we demonstrate a system for monitoring an ECG signal, one of the most basic of human bio-signals, from the subcutaneous fat of the human body.

## 2. Prototype and Demonstration of ECG Monitoring System

**Figure 1:**
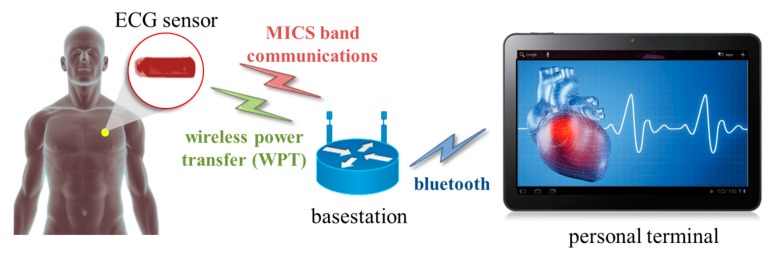
Electrocardiogram (ECG) monitoring system diagram.

Figure 1 shows a diagram of the developed prototype used to monitor ECG signals. An ECG sensor is implanted in the subcutaneous fat to sense the ECG signals and transfer the sensed signal to the basestation through the medical device radio communication service (MedRadio) band. The basestation collects the ECG signals and transfers power to the implantable device using WPT technology when the battery voltage level of the implantable device is low. A personal terminal is connected to the basestation through Bluetooth, and the user checks and monitors the status of the person with the implantable device.

**Figure 2:**
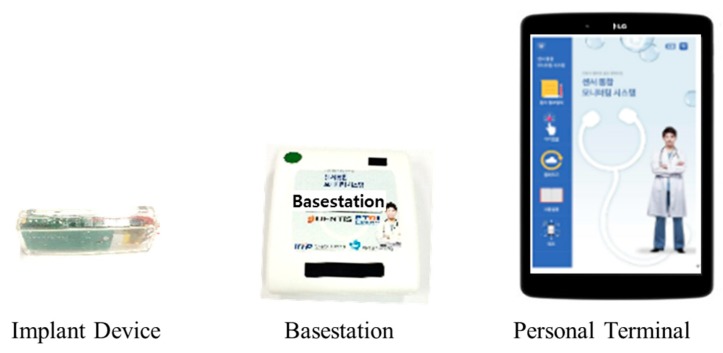
Developed prototype modules of the ECG monitoring system.

Figure 2 shows the developed prototype modules. The dimensions of the implantable device and the basestation are 19.4 mm × 55.4 mm × 9 mm and 71 mm × 77 mm × 22 mm, respectively. In the personal terminal, the developed monitoring application shows the ECG signals of the user and the temperature inside the implantable device, as well as the battery voltage level of the implantable device. 

**Figure 3:**
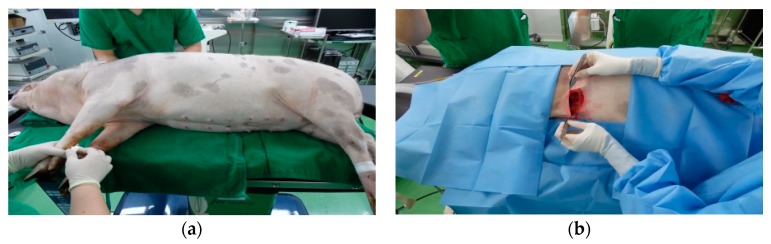
Animal experiment: (**a**) Test animal and (**b**) photograph of implanting the device into subcutaneous fat.

As shown in Figure 3, the prototype was implanted into a 40 kg Hanford (HAN) miniature pig to demonstrate its performance. Because the implantable device is small, only a small incision was required. The implantable device was implanted in the subcutaneous fat layer of the chest.

**Figure 4:**
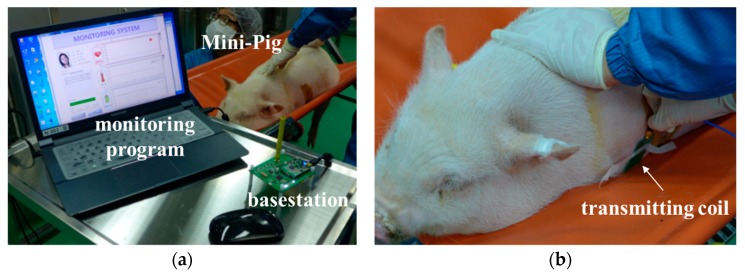
Animal experiment: (**a**) Experiment setup and (**b**) wireless charging.

**Figure 5:**
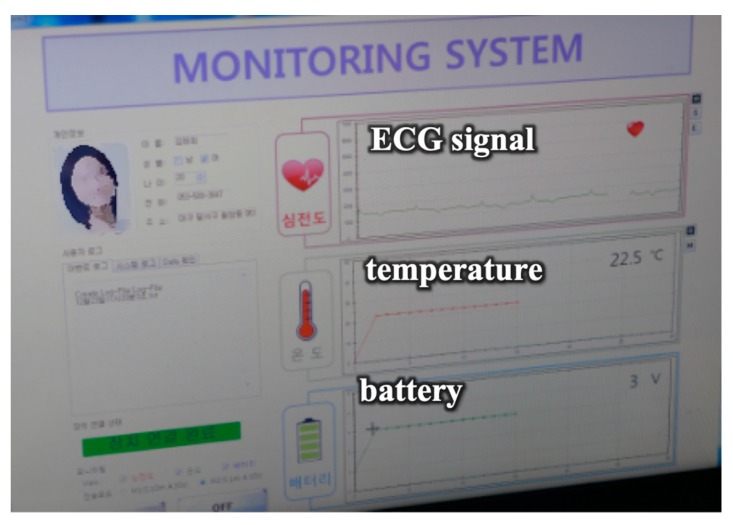
Sensed bio-signals and battery voltage level on the monitoring program.

After the device was implanted, the collected ECG signals and temperature were displayed on the monitoring program in real time, as shown in Figure 4a. In addition, power was wirelessly transferred to the implantable device from an external transmitting coil just above the skin, as shown in Figure 4b. Figure 5 shows a photograph of the monitor program displaying three types of information. As the ECG signal of the pigs differs from that of humans, the ECG signal in Figure 5 looks unusual, but works properly with periodic signals. The program displays the sensed temperature of 22.5 °C, while the body temperature of a typical pig is approximately 40 °C [14]. This discrepancy is caused by not measuring the contact body temperature but measuring the temperature inside the implantable device. Finally, we recharged the secondary cell wirelessly for 1 h after fully discharging it to check the wireless charging function. The implantable device operated normally, and the charged battery had a voltage of approximately 3 V, as shown in Figure 5.

## 3. Implantable Device

**Figure 6:**
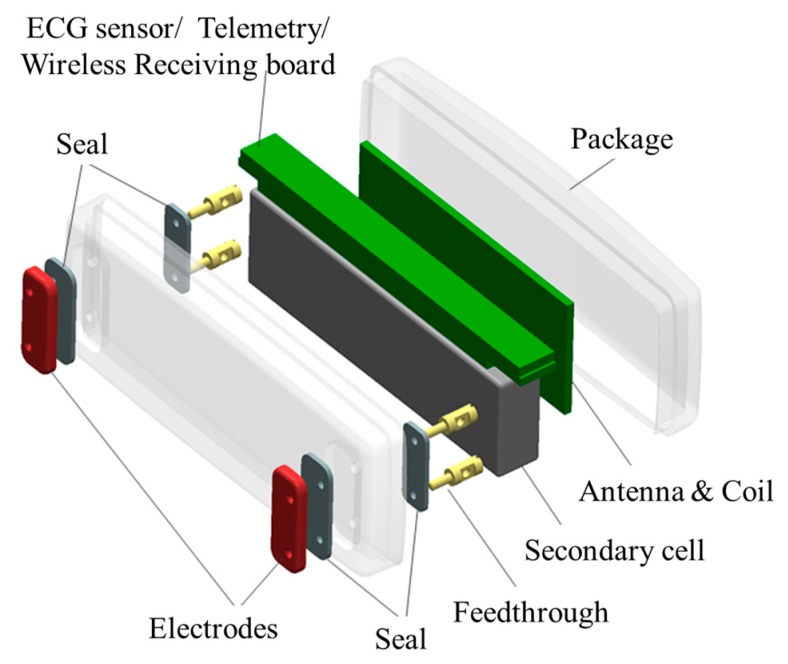
Configuration of the implantable device.

Figure 6 shows the developed implantable device consisting of a system on a board (SoB), an antenna and a coil board, a secondary cell, and a package. 

For long-term operation of the device with a single charge, a secondary cell with a large capacity is required. In general, the capacity of the secondary cell increases with its volume, and thus a cell with a large volume is required for a long operation period, which undesirably increases the dimensions of the implantable device. With the maximally optimized integration of an electric circuit on the board, the ECG sensor, telemetry and control unit, and WPT circuits, the implantable device was implemented in a single board of 50 mm × 5 mm, and the SoB was placed on the side of the secondary cell. In contrast, the antenna for the telemetry and the coil for the wireless charging should be large so as to secure a large effective area in the electromagnetic receiving ends. To this end, the antenna and coil were integrated and placed on the front side of the secondary cell. 

**Figure 7:**
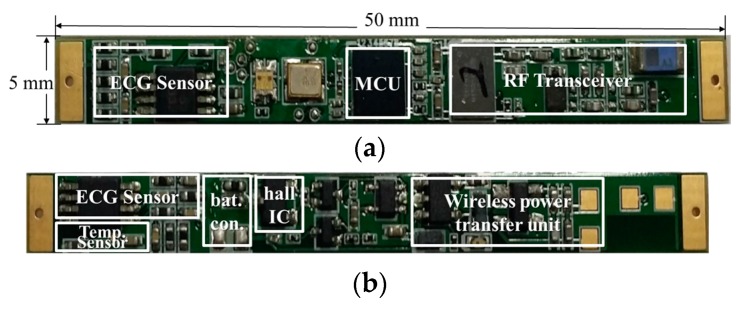
The developed system on a board (SoB) for ECG sensing, telemetry, and wireless power transfer (WPT) receiving: (**a**) Front and (**b**) back sides.

Figure 7 shows a photograph of the developed SoB upon which the ECG sensor, temperature sensor, main control unit (MCU), radio-frequency (RF) transceiver for telemetry, and WPT receiving unit are integrated. In this section, we describe these units of the implantable device in detail.

### 3.1. Sensor Unit

To capture the electric potential generated by the heart, the two electrodes must be on different equal-potential lines. This means that the distance between the two electrodes should be several centimeters or more [15]. Based on the measurement of the ECG signal according to the distance between the two electrodes, a minimum distance of 35 mm is required to obtain a stable ECG signal. Thus, the pads for connecting two electrodes were placed on both sides of the SoB, as shown in Figure 7.

**Figure 8:**
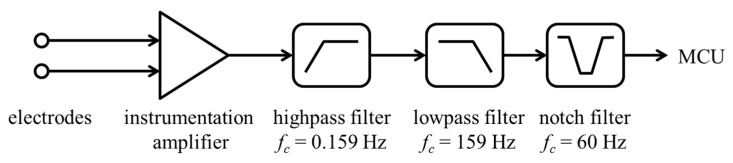
Block diagram of the ECG sensor.

The structure of the ECG sensor is shown in Figure 8. Because the heart-generated electric potential is low, within the range of 1–5 mV, the instrumentation amplifier was implemented using a TI INA333 (Texas Instruments, Austin, TX, USA) to amplify the sensed differential signals. Additionally, the high- and low-pass filters were cascaded, and the notch filter eliminated the power line noise of 60 Hz. The output of the ECG sensor entered the analog digital converter (ADC) of the MCU.

**Figure 9:**
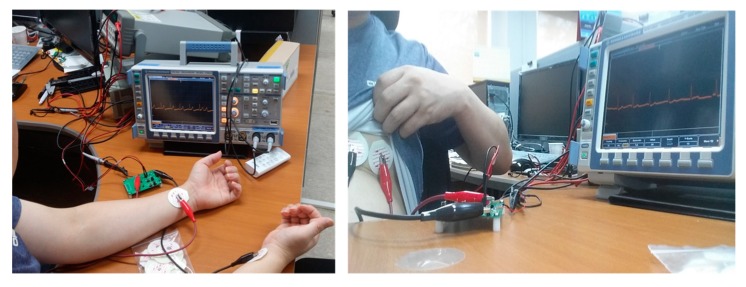
Testing the ECG signals from the wrists and chest.

At the development stage, we received the ECG signal from the sensor after attaching commercial ECG patches on the wrists or chest, as shown in Figure 9. Based on the sensed ECG signal, we tuned the cutoff frequencies of the high- and low-pass filters. The ECG signal is so weak that it is easily contaminated from a 60 Hz noise generated by surrounding power lines. Actually, a noise of 60 Hz was observed when the battery as well as power supply were powered to the sensor. Therefore, a 60 Hz notch filter was applied to the SoB. 

**Figure 10:**
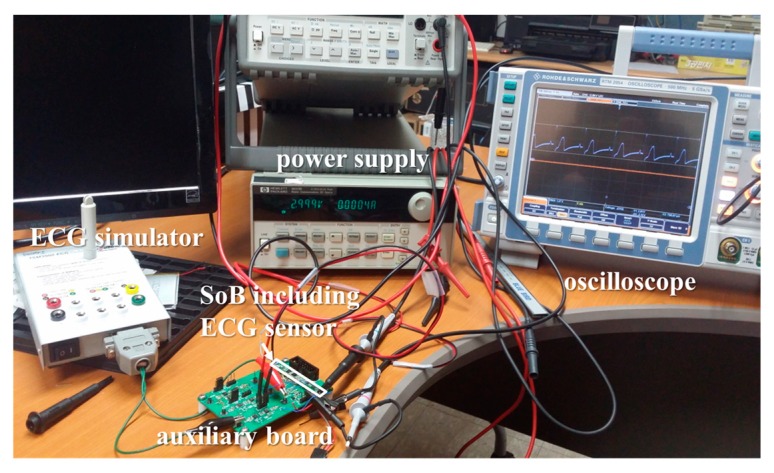
Power consumption test of the ECG sensor.

Figure 10 describes the experiment setup used to measure the power consumption of the ECG sensor module. The ECG simulator provided a virtual ECG signal, and only the parts related to the ECG sensor were turned on. For the power supply, the current provided at a supply voltage of 3 V was 0.3–0.4 mA during an active state. A Murata NCP series part was used to measure the temperature inside the implantable device, and a voltage-distributed circuit, configured for a series of resistances, was used to measure the voltage level of the secondary cell. According to the part’s datasheet, the operating temperature range was from −40 to 125 °C, but the temperature sensor was calibrated at 5-degree intervals from 20 to 40 °C, and we confirmed that the temperature sensor normally senses the right value under the circuit board. 

### 3.2. Telemetry and Control Unit

**Figure 11:**
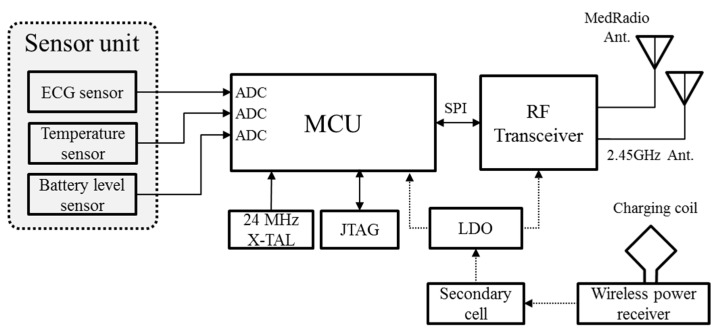
Block diagram of the SoB.

Figure 11 shows the block diagram of the SoB. The output data of the sensor units, such as the ECG, temperature, and battery voltage, were input to the ADC ports of the MCU and converted into digital data. The MCU processed and stored the imported sensing data and controled a RF transceiver as well as the sensors using the serial peripheral interface bus (SPI). The used RF transceiver and MCU were the Microsemi’s ZL70102 and the TI’s MSP430, respectively. 

Next, the converted digital data was transmitted to the basestation through the RF link. Of the various frequency bands, the MedRadio has been allocated in the 401–406 MHz for data transmission of an implanted medical device by the Federal Communication Commission (FCC) [16]. The ZL70102 transceiver operates in the MedRadio for telemetry and 2.45 GHz industrial scientific medical (ISM) band for waking up the sleep mode of the transceiver, respectively. Therefore, two antennas for MedRadio and 2.45 GHz ISM band were installed, as shown in Figure 11; a chip antenna for the 2.45 GHz frequency band and a printed antenna for the MedRadio band. The printed antenna is described in detail in the Section 3.4.

**Figure 12:**
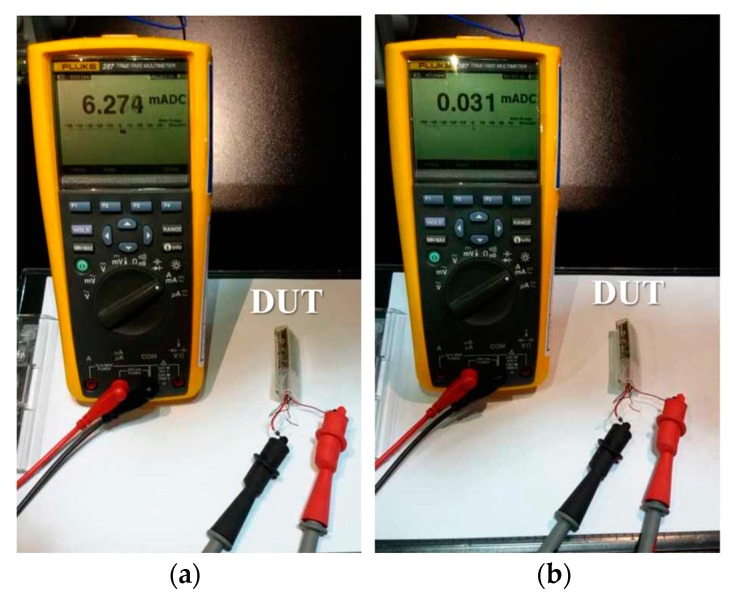
Experiments for measuring the power consumption (**a**) in the active mode and (**b**) in sleep mode.

Since the implantable device operates with limited battery capacity, low power consumption is one of the most important considerations. To measure the power consumption, we measured the DC current supplied by the secondary cell in the active and sleep modes, as shown in Figure 12. The DC rated supply voltage of the secondary cell was 3.0 V, so the estimated power consumption was 18.82 mW and 0.09 mW in the active and sleep modes, respectively. 

**Figure 13:**
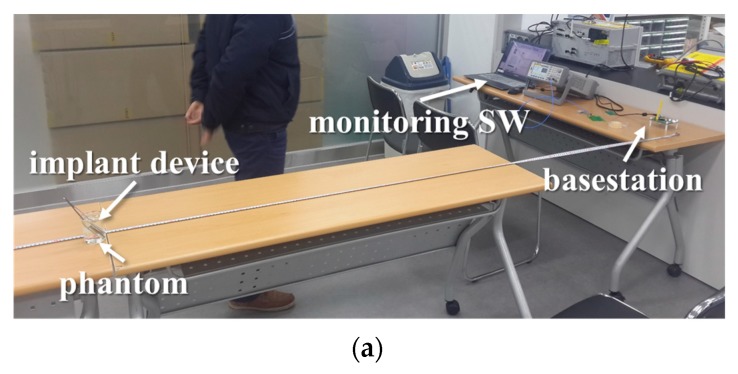

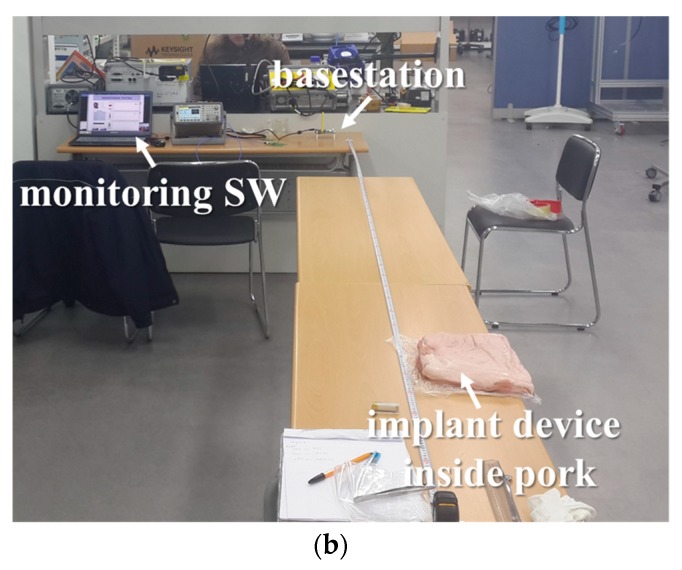
Experiment setup used to measure the maximum communication range when the implantable device is placed inside the (**a**) phantom and (**b**) pork.

The maximum communication range is another figure of merit of an implantable device. When the implantable device is on the desk, the measured maximum range was 4.2 m. However, since the implantable device should ultimately work in the human body, the communication range was measured with the implantable device located in the human body mimicking material called a phantom. As shown in Figure 13, we inserted the implantable device in the phantom and a slice of pork and measured the maximum communication range by varying the distance between the device and basestation. The experimental results confirm that the maximum communication range of the implantable device with the basestation was 2.4 m for the phantom and 2.8 m for the pork environments. 

**Figure 14:**
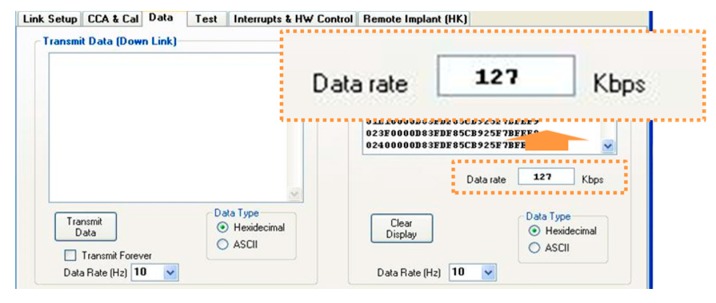
Data rate test result.

In addition, we also measured the maximum data rate from the implantable device to the basestation. Figure 14 shows that the implantable device can transmit all data, including the sensing and control signals, to the basestation at a data rate of 127 kbps. In this case, the 12-bit ADC of the MCU was operated with sampling rate of 100 Hz.

### 3.3. Wireless Power Transfer (WPT) Unit

The 13.56 MHz power signal from the receiving coil of the implantable device was converted into DC power by the WPT receiving unit, and the converted DC power was used to charge the secondary cell.

**Figure 15:**
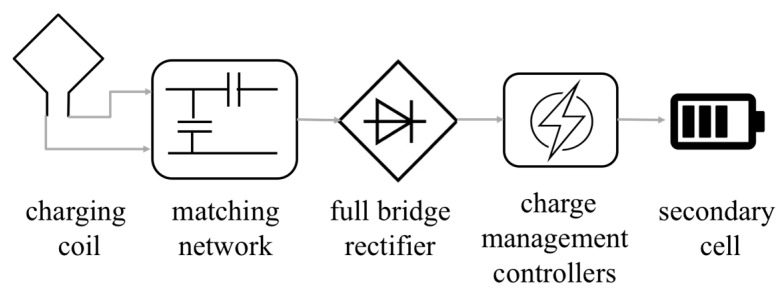
Block diagram of the WPT receiving unit.

Figure 15 shows a block diagram of the WPT receiving unit. The receiving and transmitting coils were designed to have a high Q-factor at an operating frequency of 13.56 MHz. The matching network with an L-section structure has two functions: One for generating resonance and the other for transferring the coil impedance to the input impedance of the full bridge rectifier. 

The maximum distance between the transmitting and receiving coils was about 20 mm because the implantable device was inserted into the subcutaneous fat and the transmitting coil was located on the skin. For this reason, we designed the matching network to achieve the maximum power transfer efficiency (PTE) at a distance of 20 mm. A full bridge rectifier converting AC to DC power was implemented using an Avago HSMS2828. The linear charge management controller, a Microchip MCP73831, effectively charges the secondary cell depending on the battery status, and the user can check the charge status through the LED which emits red light only when charging is in progress. The LED light can be used only for in vitro experiment, therefore, when the device is implanted in a human body, the user can check whether the device is being charged by receiving the battery voltage.

**Figure 16:**
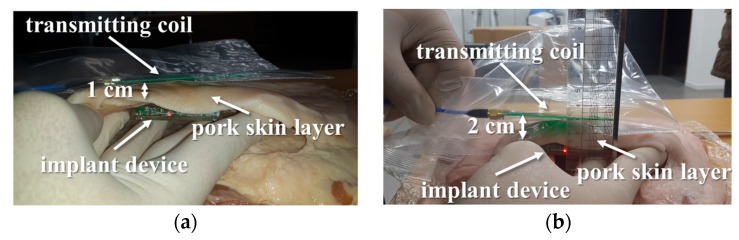
Wireless charging test in pork at distances of (**a**) 1 and (**b**) 2 cm.

**Figure 17:**
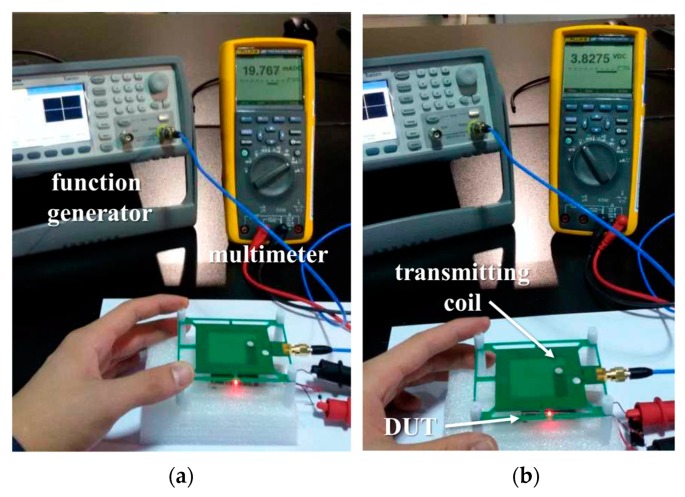
Measurement setup for (**a**) charging current and (**b**) voltage of the secondary cell.

Biological tissue does not have magnetic properties [17], and thus has little effect on the magnetic field and PTE. Nevertheless, we tested the wireless charging using pork, as shown in Figure 16. The lit LED indicates that the wireless charging function is working properly. To estimate the accurate charging power and PTE, we measured the charging current and voltage, which are the output of the linear charge management controller, as shown in Figure 17. At a distance of 20 mm, the charging power was estimated to be 75.66 mW (19.767 mA × 3.8257 V). The total PTE was also estimated to be 30.26% based on the 250 mW of maximum available power of the function generator. There is a typical trade-off between the PTE and the size of the receiving coil, and thus the larger the size of the receiving coil, the higher the PTE. 

### 3.4. Antenna for MedRadio and Coil for Wireless Charging

For the implantable device to communicate with the basestation and wirelessly charge, the antenna and receiving coil should face outward. The larger the size of the units, the higher the gain and the quality factor (Q-factor). To design a compact antenna and coil, we integrated them as described in Reference [18]. As the key feature of the design, a high-permeability sheet, such as ferrite, was introduced to the dielectric substrate to minimize the antenna and coil size and enhance the operating bandwidth of the antenna.

**Figure 18:**
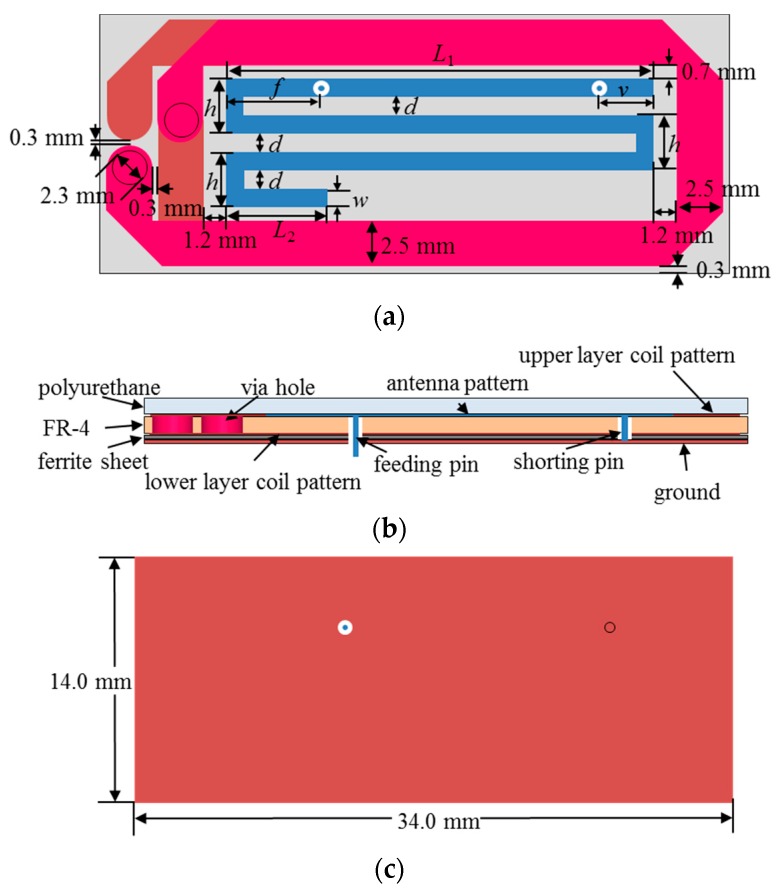
Geometry of the resonant coil and MedRadio antenna: (**a**) Top, (**b**) side, and (**c**) bottom.

The integrated coil and antenna were designed as shown in Figure 18; the antenna is inside the integrated surface, and the coil surrounds the outside of the antenna. Compared with the structure in Reference [18], the size of this version increased from 30 mm × 5 mm to 34 mm × 14 mm, making full use of the area of the secondary cell. For the coil, the number of turns was reduced from 4 to 2, and the line width widened through the optimization process. The antenna has a meandering planar inverted-F antenna (PIFA) structure, the designed parameters for which are summarized in Table 1. 

**Figure 19:**
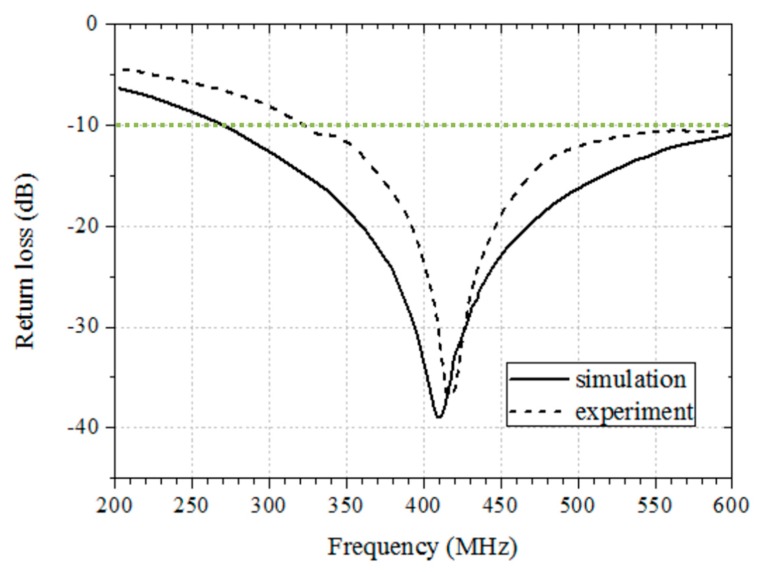
Reflection coefficient frequency responses with respect to frequency.

The characteristics of the antenna are described in detail in Reference [19]; however, the effects of the outer coil are not included. Using the phantom, we measured the return loss of the antenna with the coil, the simulation results of which are shown in Figure 19. As the simulation results indicate, the antenna has a broad bandwidth of 380 MHz with a −10 dB bandwidth criterion. However, the measured results show a bandwidth of 264 MHz, which is sufficiently wide to cover the MedRadio frequency band of 401–406 MHz.

In the simulation, the antenna showed a gain of −30 dBi at 403 MHz. Based on the regulation that the equivalent isotropic radiated power (EIRP) of the MedRadio antenna should be limited to 25 μW (−46 dBm), the input power of the antenna should be less than −16 dBm. The input power can be controlled from −30 to −1 dBm using a RF transceiver.

For the implantable antenna, the maximum specific absorption rate (SAR) is also a significant consideration. The IEEE C95.1 standard restricts the spatial SAR peak to less than 1.6 W/kg (SAR1g, max ≤ 1.6 W/kg) as averaged over any 1 g of cubic-shaped tissue [20]. According to the EM simulation, the antenna has a 1-g SAR of 39.4 W/kg (peak SAR value) when 1 W of power is delivered to the antenna, which indicates that the delivered power should be less than 40 mW. In our system, the maximum power from the RF transceiver is 1 mW, and therefore the implantable device complies with the above-mentioned SAR restriction.

The system parameters of the coil are summarized in Table 2. The Q-factor of the coil is about twice that of the previous coil described in Reference [18]. In addition, as a result of the increased size of the receiving coil, the size of the transmitting coil is also increased, and the transmitting coil has a larger Q-factor than that of the transmitting coil in Reference [18]. As a result, the efficiency of the link is increased by approximately three times. The link efficiency shown in Table 2 is defined as the efficiency between two coils, which is the efficiency excluding the influence of the low dropout (LDO) regulator in the PTE mentioned above.

### 3.5. Thermal Analysis

**Figure 20:**
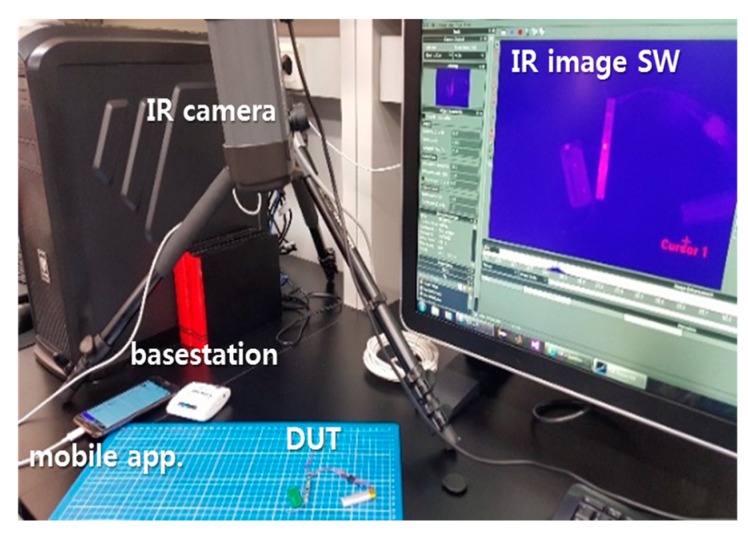
Measurement setup for measuring the temperature distribution on the SoB.

Implantable sensors are known to potentially induce inflammation of body tissues if a temperature change of more than 1 °C occurs. For this reason, the average temperature change of an implantable device should be minimized. As shown in Figure 20, we took snapshots of the temperature distribution on the SoB using an infrared (IR) camera and performed a thermal analysis. First, we distinguished the major hot spots and the parts causing hot spot, and then placed them apart. Finally, the output power of the RF transceiver was adjusted to satisfy a temperature change of 1 °C or less. 

**Figure 21:**
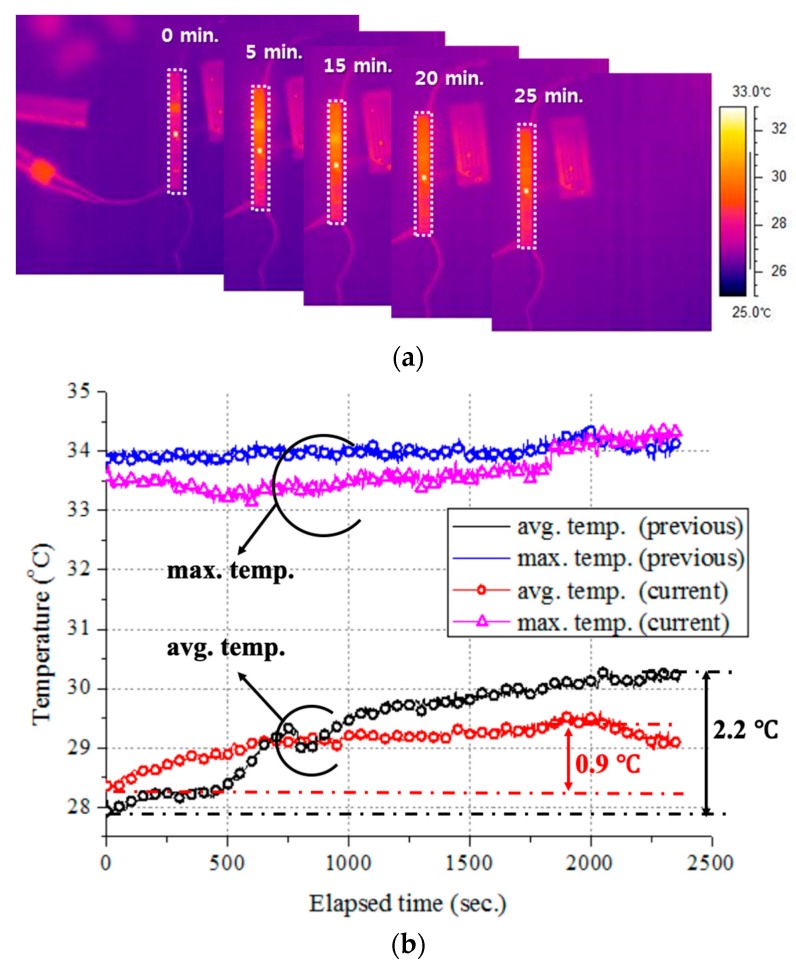
Measured thermal characteristics of the revised SoB using a thermal analysis: (**a**) Snapshots of the thermal distribution and (**b**) temperature changes.

After applying the thermal analysis results to the SoB, we observed the temperature distribution for 30 min during which the monitoring system was operating, as shown in Figure 21. The changes of average and maximum temperatures over time are shown in Figure 21b. Compared with the previous version, the maximum temperature decreased by 0.5 °C at the RF transceiver, and the change of the average temperature also decreased from 2.2 °C to 0.9 °C. Consequently, the overall temperature change in this case was less than 1 °C.

### 3.6. Battery and Package

A lithium polymer battery with a capacity of 165 mAh was used to supply the operating power. As mentioned before, the implantable device consumes 6.27 mA and 0.03 mA of current during an active and idle state, respectively. Therefore, a capacity of 165 mAh is computationally sufficient to operate the implantable device all day long without idle. In actual operation, the implantable device does not continuously detect the signal for a 24 h-period, and alternates between idle and active modes depending on the user. We set the active mode to 5 s and the idle mode to 160 s. Thus, the operating time of the implantable devicewith a single charge can be calculated from the following equation, and the calculated duration time was 31.2 days.

(1) $$165\;\mathrm{mAh}=\mathrm{Days}\times 24\;h\times \frac{6.274\;\mathrm{mA}\times 5\;s+0.031\;\mathrm{mA}\times 160\;s}{5\;s\;+160\;s}$$

However, a digital omnipolar magnetic switch (MDT MMS2X1H) connected to the LDO regulator enables the user to forcibly turn the implantable device on and off using an external magnet if necessary. This function can prevent unnecessary power consumption by turning off the power when the sensor is not used before or after putting it into the human body.

Biocompatibility is the most stringent precondition for implantable devices; the implantable device is safe to insert into the body and can operate in vivo. The biocompatibility is mainly affected by the packaging material. The most commonly used materials for biocompatible materials are cobalt-chromium, iridium, titanium, platinum, nitinol, specific glass, polydimethylsiloxane (PDMS), polyurethane, polymethylmethacrylate (PMMA), and polyimide [21]. Polyurethane with excellent electrical insulation characteristics was chosen as the packaging material. The package was sealed with ultrasonic bonding. On the other hand, electrodes are needed to measure the electric potential generated by the heart. Generally, wet electrodes such as Ag/AgCl electrodes are not suitable for long-term cardiac monitoring due to skin irritation and allergic reactions [22]. Among various dry electrodes, titanium exhibit excellent stability in vivo, so we used the electrodes fabricated using titanium.

## 4. Basestation Module

**Figure 22:**
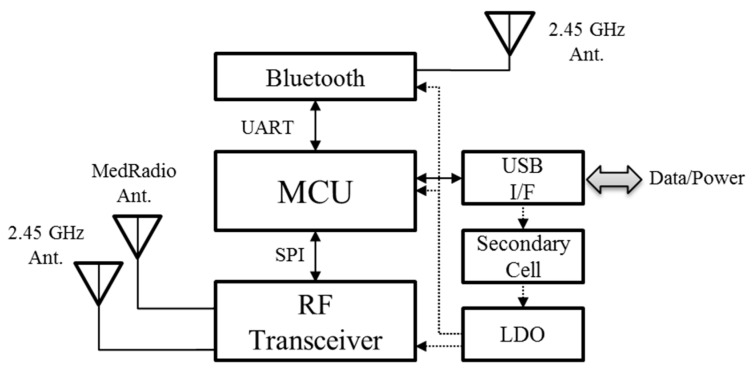
Block diagram of the basestation.

The basestation receives the bio- and control signals from the implantable device through the MedRadio band, and retransmits the signals to the user terminal via Bluetooth 4.0 low energy (BLE), as shown in Figure 22. Except for Bluetooth, most of the blocks in the basestation are similar to the SoB in the implantable device. The basestation includes LEDs indicating the charging state and connection states between the implantable device and the user terminal.

A commercial helical antenna is used for the MedRadio band, and chip antennas are used for the 2.45 GHz wake-up link and Bluetooth.

The power unit consists of a 1500 mhA lithium-polymer cell and LDOs, and users can control the power with a slide switch. A mini USB is used to charge the secondary cell through a BQ24080 charger and additionally transfer data to an external device via wire, and the charging status LED indicates whether the battery is being charged.

## 5. Conclusions

We developed an implantable ECG monitoring system and demonstrated its performance through an in vivo test using an animal. The ECG signal and temperature of the animal, as well as the voltage level of the secondary cell, were received from the implantable device and were confirmed using a PC program.

The implantable device with wireless charging technology does not require lead wires for the power supply, and can be charged even when discharged. The applied wireless charging technology shows a PTE of approximately 30%. A compact implantable device was developed by integrating a MedRadio antenna and a wireless charging coil. When implanted in pork, the developed device can communicate with the external basestation at up to a distance of 2.8 m. The implanted device consumes a current of 6.27 mA in an active state, and the adopted battery capacity is 165 mAh; therefore, it is sufficient to charge the implanted device once a month even if an ECG signal is continuously sensed. In addition, the system can be used to monitor cardiac arrhythmias and additionally manage experimental animals.

## Figures and Tables

**Table 1:** Parameters of the designed antenna.

Parameters	*L*_1_	*L*_2_	*d*	*h*	*v*	*f*
value (mm)	23.2	5.5	1	3	3	5.2

**Table 2:** Parameters and performance of the receiving coil.

Parameters	*L*	*R*	Q*-*Factor	Link Eff.
value	0.18 μH	0.316 Ω	48.48	0.61
